# Local Resource Heterogeneity Drives Density‐Dependent Dispersal Expression in a Phoretic Mite

**DOI:** 10.1002/ece3.74038

**Published:** 2026-07-30

**Authors:** Lukas H. A. Edwards, Isabel M. Smallegange

**Affiliations:** ^1^ School of Natural & Environmental Sciences Newcastle University Newcastle‐upon‐Tyne UK

**Keywords:** compensatory growth, developmental plasticity, dispersal, evolution, foraging, opportunity costs, phenotypic plasticity, resource distribution, *Rhizoglyphus robini*

## Abstract

Dispersal is favoured in heterogenous and ephemeral environments, where it allows individuals to move and access alternate patches. However, whilst heterogeneity across habitats favours dispersal evolutionarily, little is known about how resource heterogeneity at the source habitat—local heterogeneity—may cue dispersal expression. Local heterogeneity is likely important, as it may drive local competition and encourage movement around the local environment. We assessed the role of local resource heterogeneity on the expression of a plastic dispersal phenotype in experimental populations of the bulb mite *Rhizoglyphus robini* through a multigenerational experiment. Populations were kept under either heterogenous, homogenous or intermediate food distribution treatments, and at low or high population density, via culling. Thus, we tested for the effect of local resource heterogeneity and competition on dispersal expression. Additionally, we used our findings to understand the influence of compensatory growth, induced by culling, on population demography. We found that heterogenous environments induce greater dispersal expression in high‐density environments, but not in low‐density ones. Culled treatments had on average larger population sizes than other treatments, but no significant differences were found in growth rates. Relative abundance of both eggs and juveniles in comparison to adults was also significantly determined by experimental treatment, particularly culling. We infer that heterogeneity exacerbates perceived local level competition. We also propose that culled populations grow at a higher rate than non‐culled, at least in the short‐term, due to compensatory reproduction. Overall, we find a significant impact of local heterogeneity on population size and dispersal expression, much of which may arise due to the influences of heterogeneity on competition around foraging spaces. Findings highlight the pervasive effects of heterogeneity across ecological scales, and the need to distinguish between local and regional processes when modelling dispersal.

## Graphical abstract

Regional heterogeneity often selects for dispersal, as this allows individuals to move between environments. However, individuals do not experience regional conditions and must make developmental decisions based on local cues. Using long‐term population experiments, we find that local heterogeneity influences dispersal by exacerbating competition around feeding patches, cuing resource stressed individuals to attempt dispersal.

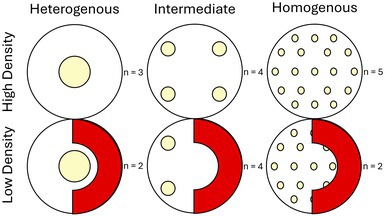

## 1 Introduction

Dispersal is a key trait that can mitigate against inbreeding, reduce competition between siblings, facilitate gene‐flow and allow the colonisation of novel habitats (Hanski and Mononen 2011). Additionally, at the individual level, dispersal can allow individuals to escape parasitism or competition for resources (Mehrparvar et al. 2013). However, dispersal is inherently risky and individuals may incur significant costs through all stages of dispersal, i.e., the pre‐departure, departure, transfer, or post‐settlement phases (Bonte et al. 2012), negatively impacting an individual's metabolism, reproductive output and survival. Given these associated costs, dispersal is predicted to occur predominantly under suboptimal environmental conditions (Chapman et al. 2011; Bonte et al. 2012) where the potential benefits of locating a more suitable habitat are likely to outweigh the fitness costs of remaining in a deteriorating environment. Heterogenous resource distributions can cause intense competition at interspersed, local feeding patches (Vahl et al. 2007; Bowler and Benton 2009; Bonte et al. 2012) and are thus an important driver of dispersal. But whilst heterogeneity in resource conditions across habitats can drive the transfer‐stage of dispersal, we know little about how fine‐scale, heterogeneity in resource conditions that are local to the source habitat drive dispersal during the pre‐departure and departure phases. Heterogeneity in local resource conditions is likely to play a critical role, as dispersal decisions are typically informed by local environmental cues, such as feeding rates and resource accessibility, which are themselves strongly influenced by the local, spatial distribution of resources (Lombaert et al. 2006; Bowler and Benton 2009; Bonte et al. 2012; Cote et al. 2017; Seeman and Evans Walter 2023). This raises questions such as how does local resource heterogeneity influence dispersal expression? What can we infer about the role of local resource distribution in competition? And what does this tell us about the role of local resource heterogeneity in the evolution of dispersal?

This study aims to quantify the role of local heterogeneity in food conditions and population density, used here as a proxy for intraspecific competition, on the facultative expression of dispersal phenotypes prior to departure. To meet this aim, we conducted a long‐term, multigenerational population experiment using the acarid bulb mite *Rhizoglyphus robini*, which has a facultative dispersive life‐stage called the ‘deutonymph’ (Diaz et al. 2000; Seeman and Evans Walter 2023). The deutonymph life‐stage is expressed just prior to the final juvenile life‐stage; it does not feed and develops morphology suited to phoresy (Diaz et al. 2000). Phoresy is a common form of dispersal among mites and other soil‐living invertebrates (Borges 2022), in which individuals attach themselves to larger organisms like arthropods, to be carried to novel habitats before detaching and metamorphosing into the later life‐stages (Borges 2022; Seeman and Evans Walter 2023). This dispersal strategy requires specialised morphological adaptations, such as a sucker plate on their dorsal side, a distinct red colouration and loss of feeding ability (Diaz et al. 2000; Borges 2022; Seeman and Evans Walter 2023). The costs of expressing these morphological adaptations include delayed development time and reduced reproductive output (Bonte et al. 2012; Deere and Smallegange 2023).

In a multigenerational experiment, we subjected 30 populations of *R. robini* to three alternate food distribution treatments and two density treatments over 18 weeks. Food distributions were either one clump of food (‘heterogenous’), four small food patches (‘intermediate’), or powdered food scattered across the population (‘homogenous’). Meanwhile, low and high‐population density was maintained through either regular culling of half the population or not‐culling populations, respectively. Importantly, all experimental populations were given the same total volume of food; thus, only the distribution of food and abundance of individuals varied between populations (Figure 2). We recorded the total number of individuals of all life‐stages and sexes, which we then used to calculate total population size, population growth rate between observations (3‐day intervals), and relative deutonymph expression. We predict that deutonymph expression (quantified as the proportion of deutonymphs against other individuals who have similarly experienced the sensitive window for this life‐stage: tritonymphs and adults) will be highest in non‐culled populations with heterogeneously distributed food because this creates the most limited access to food. Furthermore, we predict that, due to the high compensatory growth in fast life history species (Leigh and Smallegange 2014; Tanner et al. 2019), culled populations show increased reproduction resulting in increased population size, growth and relative abundance of eggs and juveniles.

## 2 Methods

### 2.1 The Bulb Mite

**FIGURE 1:**
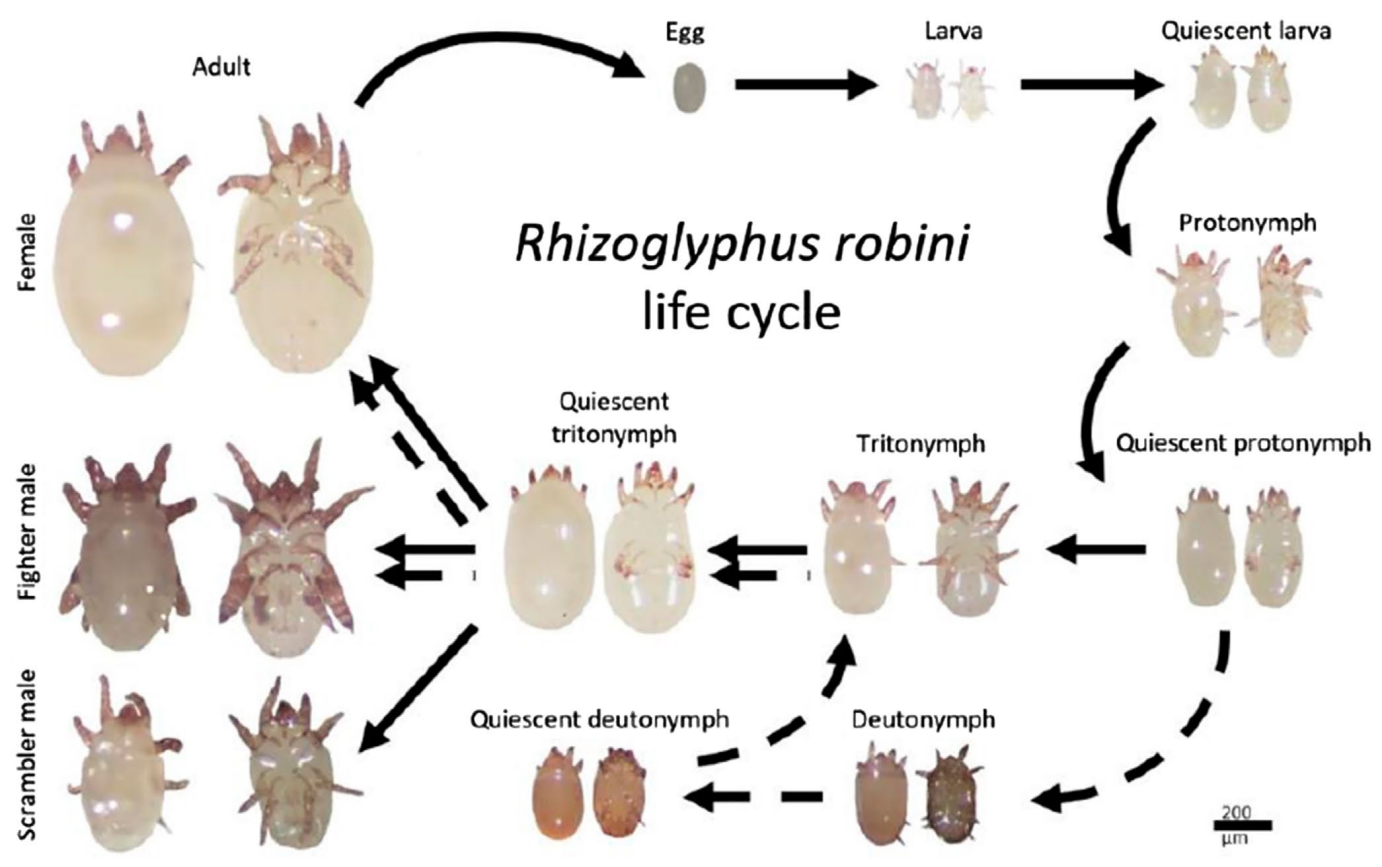
Life cycle of *Rhizoglyphus robini*. Obligatory developmental paths are shown as solid arrows, whilst plastic pathways are dashed. The lifecycle of *R. robini* has six distinct life‐stages, five of which are obligatory and one which is expressed plasticly: The deutonymph. Notably, the male morph is also a plastic trait; hence the alternate fighter and scrambler males. This figure is largely taken from Deere and Smallegange (2023), Figure 2.

*Rhizoglyphus robini* has a short lifecycle and can mature after 9–40 days (Smallegange 2011) and has a longevity between 31 and 130 days (Diaz et al. 2000). The lifecycle of *R. robini* progresses across five to six life stages: egg, larvae, protonymph, facultative deutonymph, tritonymph and adult (Figure 1; Deere and Smallegange 2023). All life‐stages except the egg and larval stage are preceded by a quiescent phase (Figure 1). Interestingly, *R. robini* has a second polyphenism, in which males may either develop into a large weaponised fighter or a small weaponless scrambler. However, this reproductive polyphenism is only visible at the adult life‐stage, which is also when one can identify the sex of individuals by their morphology.

### 2.2 Stock Cultures and Experimental Populations

*Rhizoglyphus robini* were collected from flower bulbs in a garden in Newcastle‐upon‐Tyne in June 2023. Stock cultures were subsequently established in the laboratory with *R. robini* stock cultures maintained in incubators set at 25°C. Initially, the first 3 of these stock cultures had *ad libitum* access to dried active yeast until February 2024 when another four cultures were started using individuals from the initial cultures. The new cultures were fed organic oats, a poor‐quality food source often used to induce greater deutonymph expression (Deere et al. 2015), twice a week to ensure *ad libitum* access to food. Stock cultures were watered weekly, and cultures of a food type (i.e., cultures fed on oats or cultures fed on yeast) are mixed regularly to facilitate geneflow. Oat‐fed stock cultures were allowed to acclimate to the new oat diet for 3 months prior to experiments, which equates to approximately 6 generations (Deere et al. 2015).

After acclimation, experimental populations were initiated from 100 to 200 individuals taken at random from the oat‐fed stock cultures and placed into 30 experimental, 25 mm round glass population tubes. The lower third of the population tubes was filled with plaster of Paris, which was smoothed of any air holes to prevent burrows forming and the lids of the tubes had holes drilled in which were then covered in fine mesh, to facilitate air flow but prevent escape. After transfer, experimental populations were allowed to acclimate for a further 7 weeks, approximately 3 generations (2nd May–20th June 2024), prior to experimentation. During the acclimation period, individuals were given one oat in the centre of the culture and were not culled.

### 2.3 Experimental Design

**FIGURE 2:**
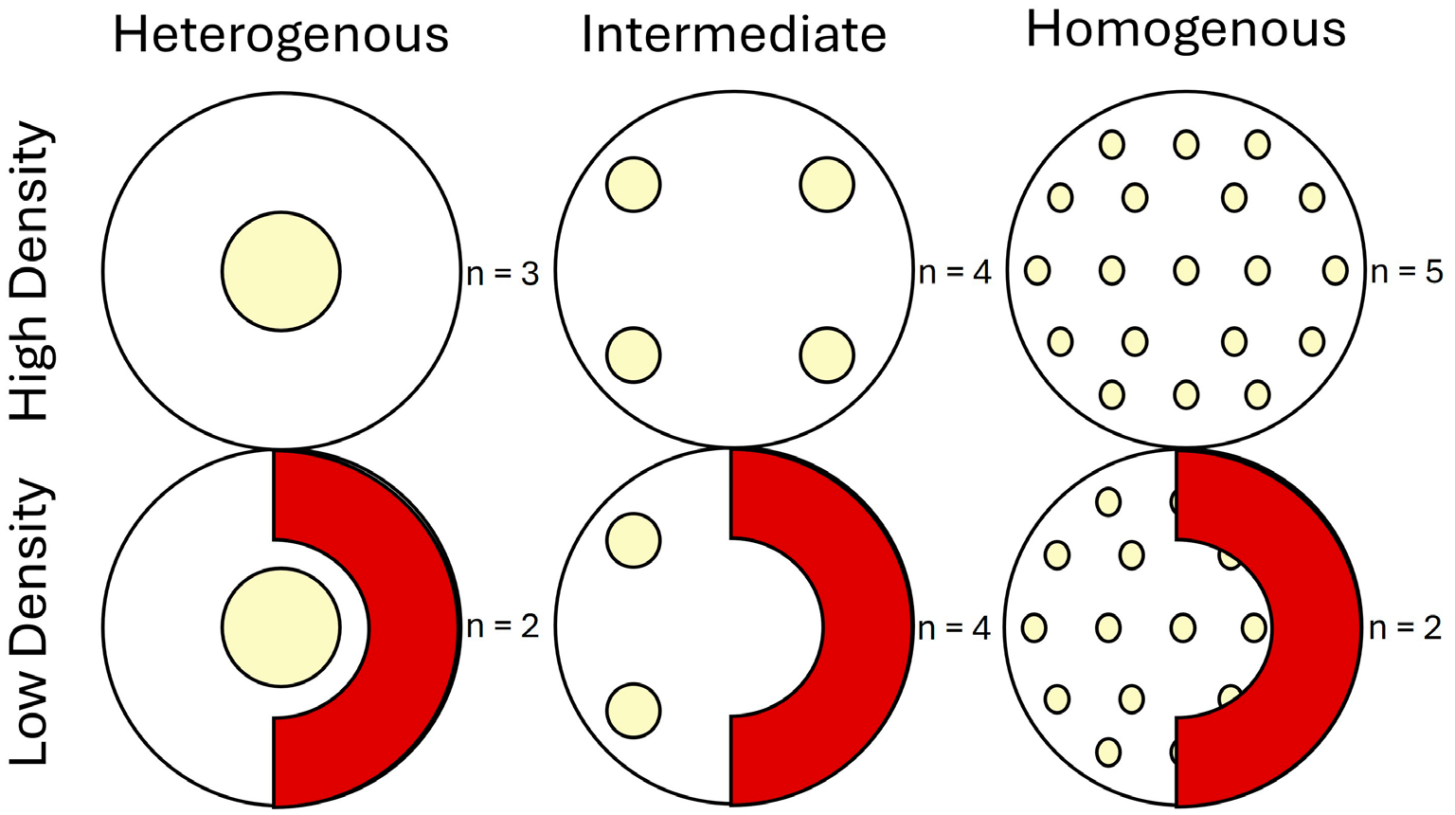
Experimental design. A third of the experimental populations were given heterogenous (one whole oat), intermediate (4 quarters of an oat), or homogenous (one oat completely crumbled/powdered) food distribution, twice a week. Half of all populations were maintained at high density, and half at low density, by culling half the population every 2 weeks. For each treatment, *n* represents the number of populations which lasted the entire duration of the study.

We started with 30 experimental populations, all of which were fed a food quantity equal to one large oat, twice a week. Ten populations were given one single, intact oat at the centre of the population (‘heterogenous’ treatment) (Figure 2). Ten populations were given a single oat but in four, equally sized quarters, one in each corner of the culture (‘intermediate’ treatment) (Figure 2). The final ten populations received a single oat that was crumbled finely and scattered evenly across the population (‘homogenous’ treatment) (Figure 2). Half of all cultures were randomly assigned to the low‐density treatment and were culled by 50% once every 2 weeks (Figure 2), after counting, whilst high‐density treatments were not culled. Treatment combinations were replicated 5 times.

We followed populations for 18 weeks, approximately 9 overlapping generations (Smallegange 2011). Populations were observed visually through a Zeiss Stemi 2000‐C microscope twice a week and the number of individuals at all life‐stages and adult sex were scored with minimal invasiveness of one quarter of each population and subsequently multiplied by four to approximate total demographics (Tom Cameron, personal communication). After counting, we conducted the culling for populations in a culled treatment, if such culling was due. Finally, we fed and watered each population. Due to logistical problems, there was a two‐and‐a‐half‐week period (week 12–14 of the experiment) where observations were not made. Additionally, we had 10 compromised populations that did not perform well or died during the experiment because they had too much or too little water, leading to mould growth or dehydration, respectively. To account for any impact on populations that experienced such excess or lack of water, but which did not experience a population collapse, we recorded population status as either 0, meaning unperturbed, or 1, meaning perturbed. Our final dataset comprised 688 observations on 20 intact populations and 10 compromised populations (Figure 2).

### 2.4 Statistical Analysis

We tested for any effects of food distribution (F; heterogenous, intermediate, homogenous), culling (C; non‐culled, culled) and time period (T; binned 3‐week intervals 1, 2, 3, 4, 5 and 6) and all their two‐ and three‐way interactions on deutonymph expression using a Generalised Linear Mixed Model (GLMM) with population tube and population status (intact or compromised) as a random factor. We used population status as a random effect since this is not expected to have a constant effect on the abundance of deutonymphs and is not a focal element of the study design. For this GLMM we used a beta‐binomial distribution to account for overdispersion in the data. To model the binomial response variable, we used the cbind function to combine the counts of deutonymphs (successes) and number of non‐deutonymphs (failures). To test our hypothesis that culling induces greater population size we used a negative binomial GLMM in which population size is predicted by food distribution, culling treatment, time period (binned 3‐week intervals), and all their interactions as fixed effects and population tube and population status as random effects on total population size. Furthermore, to assess our hypothesis that culling also influences population growth, we used a linear mixed‐effect model (LMM) to test the effects of food distribution, culling treatment, time period (binned 3‐week intervals), and all their interactions on population growth rate. Population growth was calculated by dividing population size at an observation by the population size during the previous observation. This was done using the ‘lag’ function from the tidyverse library and controlling with population id. We also included population tube as a random effect within the LMM. Additionally, we performed a natural log transformation on the population growth rates to conform to the assumption of Gaussian error distributions. Finally, to test our prediction that culled populations produce relatively more juveniles in compensation to culling, we undertook a life‐stage distribution analysis in which we quantified the influence of experimental treatment on the relative abundance of eggs and juveniles (all life‐stages preceding adulthood excluding eggs) in relation to adults. Because the relative abundances of eggs, juveniles and adults sum to unity, we applied log‐ratio transformations (Aitchison 1982), in which our response variables were the log of the proportion of eggs divided by the proportion of adults and the log of the proportion of juveniles divided by the proportion of adults. The predictors were structured the same as in other analyses, with a three‐way interaction between culling, food distribution and time, plus population tube and status as random effects within a linear mixed‐effect model using a Gaussian distribution.

### 2.5 Model Simplification

We used model simplification to acquire the best model from the full model. The least significant term i.e., the term with the highest *p*‐value, starting with the highest order interaction, was first removed from the fitted model to produce a reduced model. Model simplification proceeded by first evaluating the highest order interactions terms. Because lower order terms are required to remain in the model when included in a higher order interaction, we always began with the three‐way interaction, removing it only when non‐significant, before considering two‐way interactions and, finally, single term effects. For these models, this meant always beginning with the three‐way interaction, before moving to two‐way and finally single terms. If this removal led to a significant increase in residual deviance, we did not remove this term from the model and instead removed the second least significant term, again starting with the then highest order interaction. We tested for significant changes in deviance by using a likelihood ratio test, via an Analysis of Variance (ANOVA). If a removal did not lead to a significant increase in deviance, then this term was removed from the model, and the next least significant term of the highest order interaction was removed. We continued this likelihood ratio test procedure until all terms had been tested.

Following the removal of terms, levels within categorical variables were merged. For each categorical predictor, we merged the two least significant levels to produce a reduced categorical predictor. We then reran our model and tested this new model with our original using a likelihood ratio test, again using an ANOVA. If this merging of levels resulted in a significant increase in residual variance, then we did not merge these levels. If there was no significant increase in residual variance, then these levels were merged. We then moved to the next least significant levels of the same variable, before moving to a new variable until all levels of all variables have been tested.

After model simplification we then performed a sensitivity analysis on each random effect across all models to assess whether they accounted for a significant amount of variance. We did this by removing each random effect from the model and testing for a significant change in residual variance using a likelihood ratio test. Regardless of whether these random effects have significant or insignificant effects on the model, they are kept for robustness. Where these sensitivity analyses suggested model singularity, this was further tested by using the *check_singularity* function from the performance package. When such singularity is induced by random effects, it is reported. All analyses and model reduction were carried out using R (v4.3.0) (R Core Team 2023).

## 3 Results

### 3.1 Deutonymph Expression

**FIGURE 3:**
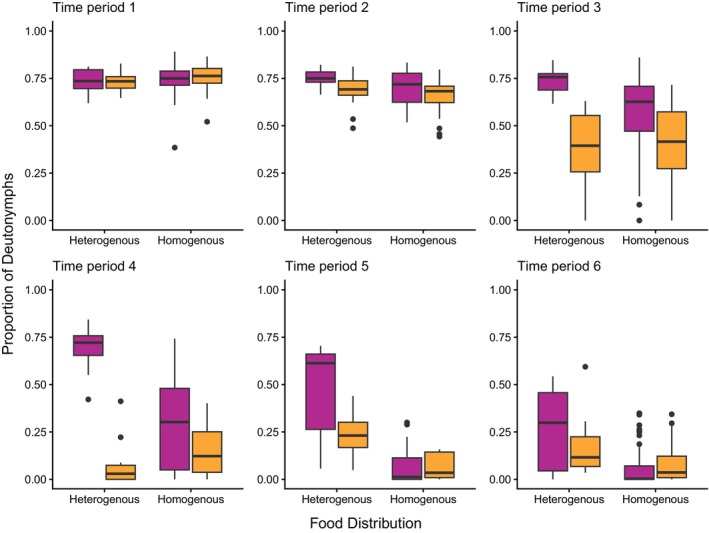
The effect of culling and resource heterogeneity on the proportion of deutonymphs across time. Across time‐blocks the proportion of deutonymphs within populations decreases significantly, with the earliest responses being in culled populations (orange) and slower responses from non‐culled populations (green). Culled and non‐heterogenous environments cue reduced plastic expression of deutonymphs, compared to the non‐culled heterogenous environments.

Deutonymph expression was significantly affected by the three‐way interaction between food distribution, culling and time period (T) ($\chi_{10}^{2}$ = 47.11, *p* < 0.001). Within this interaction, deutonymph expression in the ‘intermediate’ and ‘homogenous’ food distributions did not significantly differ ($\chi_{2}^{2}$ = 1.85, *p* = 0.3975) and were combined into one level, which we called ‘non‐heterogenous’ food distribution. The model accounted for 91.7% of the variance, of which 73.6% is explained by fixed effects and 18.1% by random effects. Of the variance explained by random effects, 2.7% is from population ID and 15.4% is from population status. We also found that both population tube ($\chi_{2}^{2}$ = 68.486, *p* = < 0.001) and population status ($\chi_{2}^{2}$ = 54.954, *p* = < 0.001) accounted for a significant amount of variance in the model. The overall result was that the relative abundance of deutonymphs is lowest under non‐heterogenous resource distributions or low population density but is highest when resources are heterogeneously distributed and population density is high (Figure 3). However, these interactions are much more pronounced during later timesteps, from T4 onwards, and there are no significant impacts of experimental treatment during timestep 1 or 2.

### 3.2 Population Size

**FIGURE 4:**
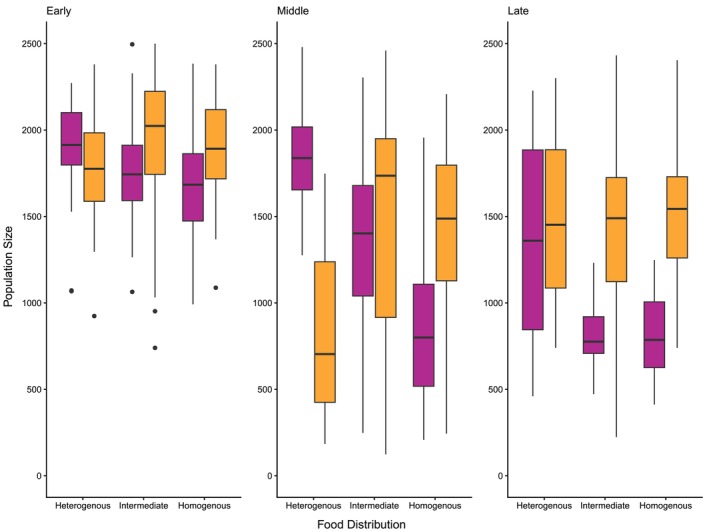
The effect of food distribution, density and time on population size. Non‐culled populations (green) generally have lower population sizes, whilst culled populations (orange) have higher. Despite this, populations which are both high density and have clumped food distributions have large population sizes, greater than low density populations in Middle time periods and on par with low density populations during late time periods. No significant differences were seen in the early time step since populations were likely in the process of responding to cues.

Population size is significantly influenced by the three‐way interaction of food distribution, culling and time period ($\chi_{10}^{2}$ = 36.13, *p* < 0.001). Within time‐period we found that T1 and T2 ($\chi_{2}^{2}$ = 2.71, *p* = 0.844), T3 and T4 ($\chi_{2}^{2}$ = 14.35, *p* = 0.279), and T5 and T6 ($\chi_{2}^{2}$ = 18.55, *p* = 0.420) could be merged without significantly increasing the deviance of the model. This creates three time periods, ‘early’, ‘middle’ and ‘late’, which corresponds to weeks 1–6, 7–12 and 13–18, respectively. The sensitivity analysis also showed that both population tube ($\chi_{2}^{2}$ = 65.91, *p* = < 0.001) and population status ($\chi_{2}^{2}$ = 193.92, *p* = < 0.001) accounted for a significant proportion of the variance. This GLMM explains 74.5% of the variance, of which the majority (52.9%) is explained by the random effects, and only 21.6% is explained by fixed effects. Of the variance explained by the random effects, 5% is explained by population tube and 47.9% is explained by population status. Whilst non‐culled populations are surprisingly smaller than culled populations (Figure 4), populations which are both non‐culled and have a clumped food distribution maintain a higher population size than other food distributions (Figure 4). This is not the case for culled populations, which have generally equal populations sizes, and food distribution only influences this during the middle timestep in which clumped foo distributions lead to smaller population sizes (Figure 4). Importantly, there are no significant effects or interactions from the early timestep.

### 3.3 Population Growth Rate

Population growth is not significantly influenced by food distribution ($\chi_{2}^{2}$ = 0.49, *p* = 0.784) or population size ($\chi_{2}^{2}$ = 1.22, *p* = 0.269). However, population growth is significantly influenced by time ($\chi_{2}^{2}$ = 26.43, *p* = > 0.001). Specifically, in time period 3, population growth was low (mean growth = −0.108 ± 0.044), whilst population growth was high in time period 4 (mean growth = 0.117 ± 0.044) whereas population growth rate in all other time periods did not differ from each other ($\chi_{2}^{2}$ = 23.587, *p* = 0.886, mean growth = −0.061 ± 0.063). However, the strength of this effect is very weak and only accounts for 4% of the variance in the model. Population status accounted for a further, non‐significant 5.6% of the variance ($\chi_{2}^{2}$ = 1.320, *p* = 0.251) and population tube was also not significant ($\chi_{2}^{2}$ = 0, *p* = 1), while accounting for 0% of variance and inducing singularity.

### 3.4 Life Stage Distributions

#### 3.4.1 Eggs

**FIGURE 5:**
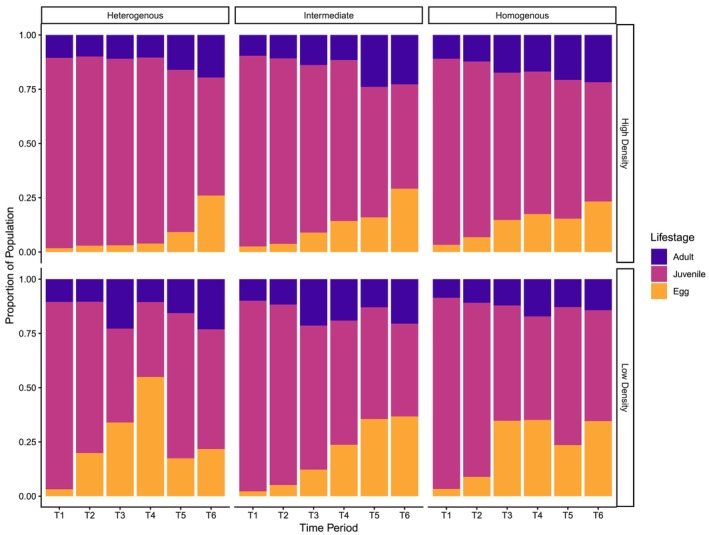
Relative abundance of life‐stages across time and treatments. This figure shows the relative abundance of each life‐stage as a proportion of the overall population. Low‐ and high‐density treatments, corresponding to culled and non‐culled respectively, are shown on the y axis, and each resource distribution treatment is shown on the x axis. Within each treatment, bars show the mean abundance of each life‐stage across a 3‐week period within each of those treatment combinations.

The relative abundance of eggs against adults is driven by a three‐way interaction between culling, food distribution and time ($\chi_{2}^{2}$ = 44.49, *p* < 0.001) (Figure 5). However, the effects of homogenous and intermediate food distributions were not significantly different ($\chi_{2}^{2}$ = 15.82, *p* = 0.200), so these levels were merged, as were time‐periods 5 and 6 ($\chi_{2}^{2}$ = 6.02, *p* = 0.197). Sensitivity analyses shows that while population pot is important to the model ($\chi_{2}^{2}$ = 59.28, *p* < 0.001), population status is not ($\chi_{2}^{2}$ = 0.55, *p* = 0.46). This model accounted for 49.7% of variance, 39.4% of which came from fixed effects and a further 10.3% from random effects. From the random effects, 9.9% is explained by population pot and a further 0.4% is explained by the non‐significant population status.

#### 3.4.2 Juveniles

The relative abundance of juveniles against adults is influenced by the two‐way interactions between culling and distribution ($\chi_{2}^{2}$ = 5.34, *p* = 0.069), culling and time ($\chi_{2}^{2}$ = 29.81, *p* < 0.001) and time and distribution ($\chi_{2}^{2}$ = 59.39, *p* < 0.001) (Figure 5). This is also significantly influenced by both population pot ($\chi_{2}^{2}$ = 20.46, *p* < 0.001) and status ($\chi_{2}^{2}$ = 20.46, *p* < 0.001). The model accounts for 59.2% of variance in relative juvenile abundance, 33.7% from fixed effects and 25.5% from random effects. From random effects, 3.8% of variance was driven by population pot and 21.7% by population status.

## 4 Discussion

### 4.1 Dispersal Expression

We explored the role of local, rather than regional, resource heterogeneity in influencing dispersal expression in *R. robini*. In line with our predictions, we found that fine‐scale local heterogeneity increased deutonymph expression. One explanation for this is that heterogenous food distributions cause reduced feeding space around patches, which in turn reduces accessibility due to competition for feeding space (Jambhekar and Isvaran 2021; Trevail et al. 2019). Although not resulting in starvation, restricted access to food could reduce feeding frequency, which serves as a cue in the regulation of other plastic traits across taxa (e.g., the nematode: Casasa et al. 2021; spadefoot toad: Storz 2004). Such individuals experience physiological stress due to unpredictable foraging opportunities and intermittent periods of starvation yet retain sufficient energy reserves to support adaptive developmental plasticity (Massot and Clobert 1995; Bonte et al. 2012; Cote et al. 2017). Indeed, such food insecure individuals may experience increased physiological stress and could be cued to store a greater proportion of their energy intake to sustain them during periods of poor food access, the so‐called “insurance hypothesis” (Nettle et al. 2017). There is also a further potential role of heterogeneity in that is encourages crowding in limited feeding areas, intensifying mechanosensory and hormonal cues from conspecifics. Such cues play a pivotal role in other dispersive polyphenisms, such as in desert locusts where regular tactile stimulation on the hind femur caused ‘gregarization’ over 4 h (Rogers et al. 2003) and in pea aphids, where tactile cues of crowding cause greater volancy in offspring (Brisson et al. 2026). The role of heterogeneity in intensifying perceived competition is further evidenced by the interaction between food distribution and population density; specifically, under non‐culled, heterogenous environments having the highest proportion of dispersers. Our results indicate that heterogenous resource distribution can drive dispersal by promoting crowding around feedings sites, due to limited feeding area. Such crowding may cause greater perceived competition (Rhebergen and Smallegange 2021), arising from increased physical and pheromonal cues from conspecifics, and realised competition arising from competition for feeding space reducing the regularity of food access.

Interestingly, we found that deutonymph expression reduced across all treatments over the course of the experiment. In our experiment, deutonymphs were unable to disperse from their cultures. Deutonymph expression is costly in terms of morphology development, longer maturation time and reduced reproductive output (Deere et al. 2015), potentially making it maladaptive in this context and reducing its expression (Ghalambor et al. 2015). Counter to our findings, Deere and Smallegange (2023) previously found that when deutonymphs were selectively culled, expression of this stage increased across the population, with only minor reduction in deutonymph expression late into their experiment. The driver of such a difference likely comes from the age of the stock cultures being used, with ours being approximately 1 year old and those of the previous study being 6 years old (Deere and Smallegange 2023). Furthermore, the bulbs from which our mites were collected had been recently planted, suggesting that the resident mite population may represent a ‘frontier’ population, characterised by elevated dispersal propensity (Simmons and Thomas 2004), which is particularly evident in the markedly higher initial proportion of deutonymphs observed in our study (~75%) compared to the ~0.7% reported by Deere and Smallegange (2023). It is likely then, that the mites used by Deere and Smallegange (2023) had adapted to laboratory settings such that the rate of deutonymph expression was already low, whilst our populations were still undergoing this adaptive process. Whilst not a confounding factor, this underscores the importance of considering the evolutionary trajectory of laboratory populations when investigating evolutionary processes, since population age, a proxy for their evolutionary distance from ‘natural’ populations, may strongly influence trait development.

To examine heterogeneity and dispersal in natural settings, the influence of habitat fragmentation and deterioration could be good proxies. For example, habitat fragmentation significantly reduces connectivity and geneflow by increasing dispersal distance through non‐feeding environments. However, if an area is locally fragmented, i.e., foraging patches are often bounded by easily traversable non‐foraging areas, then this could be a driver for the expression of dispersal characteristics. Again, pea aphids are a model example for this, since although individuals can walk between neighbouring plants to explore novel hosts high degrees of perceived density will induce wing development in offspring, which will in turn be able to travel much further than their walking conspecifics (Xu et al. 2011; Ben‐Ari et al. 2015; Brisson et al. 2026). Habitat deterioration of environments can also be a key driver for dispersal, as is seen across model systems (Pfennig 1992; Renahan et al. 2021; Seeman and Evans Walter 2023). Specifically, if the primary niche of a species is prone to deterioration, then dispersal is likely to evolve. For example, due to rising global temperatures small water bodies may be prone to drying creating a selective pressure for rapid growth and dispersal among their inhabitants, as is seen among Mexican spadefoot toads, *Spea multiplicate* (Pfennig 1992; Pfennig and Martin 2009). Given the role of fragmentation and deterioration in the evolution of dispersal, the Anthropocene is likely to induce a high selective pressure for dispersal through land‐use change and global warming. However, the challenge is in that the strength of these pressures may be practically too large to adapt to and are more likely to drive species to extinction.

To conclude this section, while broader ecological contexts are important for the sustained expression of dispersal traits, the local environment is pivotal in driving their initial expression. Individuals are generally unable to predict conditions in alternate environments and must thus rely on local cues to determine the expression of dispersal. Local resource heterogeneity is an important component of this, influencing the intensity of competition and mechanosensory or pheromonal cues from conspecifics. Thus, heterogeneity is a driver for dispersal across scales, influencing its expression at the local scale and adaptiveness at regional scales.

### 4.2 Impact of Local Environment Structure on Population Dynamics

Despite our finding that population size decreased over time, we did not observe a corresponding change in population growth. However, even if population growth rate did not statistically differ between treatments, small differences in population size may compound over time, potentially explaining why we observed significant differences in population size. Interestingly, population size was on average higher for culled populations than for the unculled controls, likely because of compensatory growth in response to culling, as seen before (e.g., Smallegange and Ens 2018). Indeed, culled treatments had a greater relative abundance of eggs compared to adults, almost regardless of food distribution. This compensatory impact of culling was less pronounced for the relative abundance of other juvenile life‐stages, which were driven more by food distribution. In natural populations, compensatory growth could have distinct implications for dispersal expression since populations often ‘over‐shoot’ their carrying capacity, leading to resource limitation and increased mortality (Leigh and Smallegange 2014; Tanner et al. 2019). While it did not happen in our study, likely due to continuous culling and feeding that reduced resource depletion, compensatory growth and dispersal could prompt an ‘eco‐phenotypic dynamic’ (Edwards and Smallegange 2025), in which low density cues compensatory growth, increasing density and cueing dispersal expression. Such an eco‐phenotypic dynamic is prevalent across other model systems expressing plastic dispersal. Pea aphids are a good example of this, where colonisation is followed by rapid asexual reproduction and population growth until resources become limited, and individuals begin dispersing to alternate host plants (Xu et al. 2011). A further example can be seen in the nematode *Pristionchus pacificus* in which a distinct boom‐and‐bust cycle is observed as population deplete their food supply and enter the dauer stage until it recovers (Renahan et al. 2021). The dispersal traits of all these systems, much like compensatory growth, likely arises as an adaption to rapidly changing or deteriorating environments. This is especially notable given all three of these examples express the same broad pattern of eco‐phenotypic dynamic, regarding their growth and subsequent dispersal (Edwards and Smallegange 2025). One exception to the trend of greater population size in culled populations is that populations on heterogenous food distributions maintained the highest population size among the unculled treatments, reaching levels comparable to those observed in the culled treatments. The primary age‐ difference between this and other non‐culled treatments is the disproportionate abundance of deutonymphs. The abundance of slower developing, non‐feeding individuals may inflate population size since these individuals will not need to compete as strongly for resources compared to non‐dispersive individuals. Thus, even fine‐scale environmental structure can shape key life history traits, underscoring the importance of incorporating local ecological context into studies of dispersal.

In our analyses we included population status as a random effect, which often explained a significant proportion of the variance. For example, it accounted for over 50% of the variance in population size. This may reflect the impact of environmental stressors such as water scarcity, which reduces survival and supresses population growth. However, population status also explained 18% of the variance in deutonymph expression, which is less easily attributed to direct effects on population size. One reason may be that deutonymph expression requires significant energetic investment and individuals experiencing physiological stress, such as dehydration, may be too stressed or lack the resources to develop this costly trait. Indeed, individuals that express the deutonymph stage suffer reduced size at maturity and lower reproductive success, highlighting the substantial life history costs associated with dispersal in this species (Deere et al. 2015). Alternatively, population status may influence deutonymph indirectly via density‐dependent effects: in stressed populations, mortality among large‐bodied individuals may be high and reproduction may decline (e.g., Smallegange and Deere 2014), altering the conditions under which deutonymph development is triggered. These findings highlight how physiological condition and population density can interact in complex ways to shape not only developmental outcomes like dispersal morph expression, but also broader ecological dynamics (Oyama et al. 2001; Edwards and Smallegange 2025; Smallegange and Guenther 2025).

An interesting feature of the results is the time lag before significant trends in deutonymph expression are observed. The most likely explanation for this is that deutonymph expression is a form of developmental plasticity that can only be initiated during juvenile stages. While *R. robini* can mature in as little as 13 days, adult females can live for 3 months (Smallegange 2011), and the deutonymph stage itself can further extend development time (Deere et al. 2015). It is therefore likely that early in our experiment, a substantial proportion of individuals were already adults that had developed prior to the onset of experimental treatments. This would delay any population‐level response in deutonymph expression, as only new juveniles would be capable of expressing the trait. Moreover, the energetic costs and developmental constraints associated with deutonymph development (Deere et al. 2015) may further restrict its expression to specific windows of ontogeny, reinforcing the importance of timing in plastic trait responses. These dynamics highlight the importance of considering life history schedules when interpreting plasticity trait responses in experimental systems. Indeed, as Walasek et al. (2024) argue, the lag between environmental change and phenotypic response is tightly linked to generation time, meaning that even short‐lived species may exhibit delayed plastic responses if trait expression is developmentally constrained. Understanding these developmental constraints is essential for predicting how populations respond to environmental change, as they mediate the pace and nature of eco‐evolutionary feedbacks.

### 4.3 Concluding Remarks

We propose that local heterogeneity, such as uneven food distribution, can be a prominent driver of dispersal expression alongside regional heterogeneity. Whilst we find that heterogenous resource distributions cue dispersal expression, they only do so under high population density. This suggests the decision to disperse may be driven by competition and resource stress, as previous work has suggested, which in turn is largely determined by resource distribution. Furthermore, a key message of this paper is that we should distinguish between spatial scales when assessing the drivers of traits, since the mechanisms by which traits are expressed, for example through selection or plasticity, will be different across these scales. By identifying how local environmental structure interacts with density to shape plastic trait expression, our findings contribute to a more mechanistic understanding of how ecological pressures translate into eco‐evolutionary responses within populations.

## Author Contributions

**Isabel M. Smallegange:** conceptualization (supporting), formal analysis (supporting), investigation (supporting), methodology (supporting), supervision (lead), writing – review and editing (equal). **Lukas H. A. Edwards:** conceptualization (lead), formal analysis (lead), investigation (lead), methodology (lead), validation (lead), visualization (lead), writing – original draft (lead), writing – review and editing (equal).

## Conflicts of Interest

The authors declare no conflicts of interest.

## Data Availability

The data and R scripts used in this study are available on figshare (https://doi.org/10.6084/m9.figshare.29665871). A public preprint of this study is also available at: https://doi.org/10.1101/2025.07.29.667385.
